# Stem cell-derived exosome delivery systems for treating atherosclerosis: The new frontier of stem cell therapy

**DOI:** 10.1016/j.mtbio.2024.101440

**Published:** 2024-12-30

**Authors:** Hassan Tariq, Syeda Zunaira Bukhari, Ruibing An, Jian Dong, Ayesha Ihsan, Muhammad Rizwan Younis

**Affiliations:** a Institute of Optical Functional Materials for Biomedical Imaging, School of Chemistry and Pharmaceutical Engineering, Shandong First Medical University & Shandong Academy of Medical Science, Taian, Shandong, 271016, PR China; b Department of Chemical and Biomolecular Engineering, University of California - Los Angeles, Los Angeles, CA, 90095, USA; c National Institute for Biotechnology and Genetic Engineering College, Pakistan Institute of Engineering and Applied Sciences (NIBGE-C, PIEAS), Faisalabad, Pakistan; d Department of Molecular, Cell and Developmental Biology, University of California - Los Angeles, Los Angeles, CA, 90095, USA

## Abstract

Cardiovascular diseases (CVDs) are a leading cause of mortality worldwide. As a chronic inflammatory disease with a complicated pathophysiology marked by abnormal lipid metabolism and arterial plaque formation, atherosclerosis is a major contributor to CVDs and can induce abrupt cardiac events. The discovery of exosomes' role in intercellular communication has sparked a great deal of interest in them recently. Exosomes are involved in strategic phases of the onset and development of atherosclerosis because they have been identified to control pathophysiologic pathways including inflammation, angiogenesis, or senescence. This review investigates the potential role of stem cell-derived exosomes in atherosclerosis management. We briefly introduced atherosclerosis and stem cell therapy including stem cell-derived exosomes. The biogenesis of exosomes along with their secretion and isolation have been elaborated. The design engineering of exosomes has been summarized to present how drug loading and surface modification with targeting ligands can improve the therapeutic and targeting capacity of exosomes, demonstrating atheroprotective action. Moreover, the mechanism of action (endothelial dysfunction, reduction of dyslipidemia, macrophage polarization, vascular calcification, and angiogenesis) of drug-loaded exosomes to treat atherosclerosis has been discussed in detail. In the end, a comparative and balanced viewpoint has been given regarding the current challenges and potential solutions to advance exosome engineering for cardiovascular therapeutic applications.

## Graphical abstract

**Figure:**
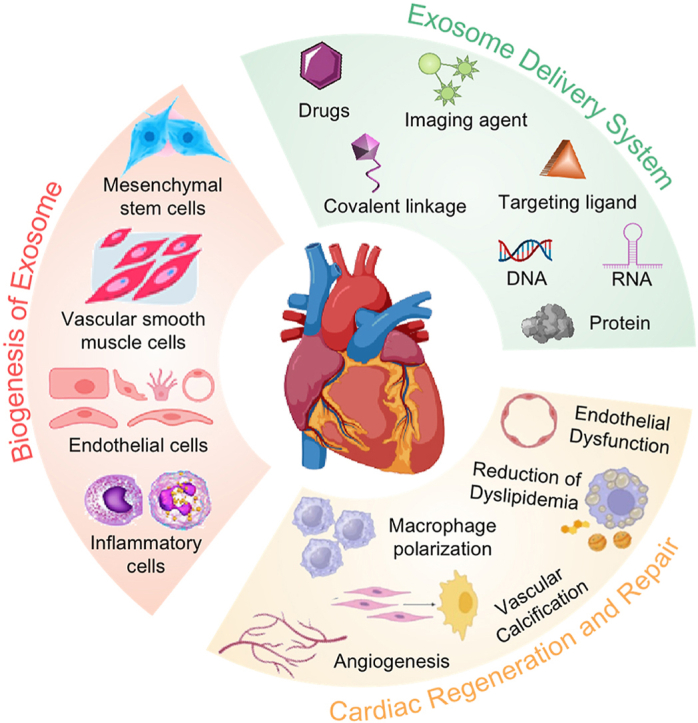

## 1 Introduction

With an estimated 17.6 million deaths annually, cardiovascular diseases (CVDs) account for one-third of the total deaths worldwide [1]. Atherosclerosis, the major cause of CVDs, is a chronic inflammatory condition with a buildup of cholesterol-based plaques in the arteries. These plaques can narrow or block the elastic and musculoelastic arteries, resulting in the development of various CVDs such as coronary heart disease, stroke, aortic aneurysm, and gangrene [2]. Atherosclerosis has become a major cause of death among the population due to the expanding global economy, the adoption of poor dietary habits, and increased environmental pollution [3]. The enormous global burden of CVD, which experts predict will increase substantially, particularly in low- and middle-income countries, cannot be addressed by improved patient care alone. This realization is the reason for the increasing focus on CVD prevention [4]. Thus, atherosclerosis management should address all known treatable risk factors due to its multifactorial nature. Reducing cholesterol, blood pressure, and tobacco use has significantly reduced CVD mortality in many populations, but lifestyle factors like obesity, type 2 diabetes, sedentary behavior, and psychosocial stress challenge these positive results [5]. Furthermore, both experimental and clinical evidence indicate that inflammation plays a crucial role in developing atherosclerotic events [6]. Recent clinical trials have demonstrated that reducing inflammation can lower atherosclerotic events even in patients who have previously undergone complete efficacious conventional treatments [7].

Mesenchymal stem cells (MSCs) are known to exhibit substantial immunomodulatory capabilities. Animal studies and human trials have provided evidence that MSCs possess the ability to suppress immunological and inflammatory responses in tissues, resulting in favorable treatment outcomes for a range of diseases [8,9]. Stem cell therapy can reportedly treat atherosclerosis by enhancing endothelial function, restoring damaged endothelium, and inhibiting plaque formation [10]. However, maintaining the transplanted cells at the target tissue or organ is difficult due to their post-transplant dispersion. Moreover, transplanted cells require consistent phenotypic cell supply due to low survival rates in ischemic conditions. This hinders their differentiation into cardio-progenitor cells (CPCs) as they transition from in vitro to in vivo environments. Also, the process always carries the risk of host immune rejection [11]. Thus, it may not be the most effective option for long-term treatment, particularly after the engrafted cells are removed. Multimodal therapy may be required to achieve long-term treatment [12]. However, small extracellular vesicles (EVs), also known as exosomes, present an innovative acellular method that effectively addresses the limitations of direct cell therapy [13]. Specifically, EVs produced by MSCs offer significant advantages over whole-cell treatment, including low immunogenicity, ease of storage, and enhanced biosafety [14].

**Scheme 1:**
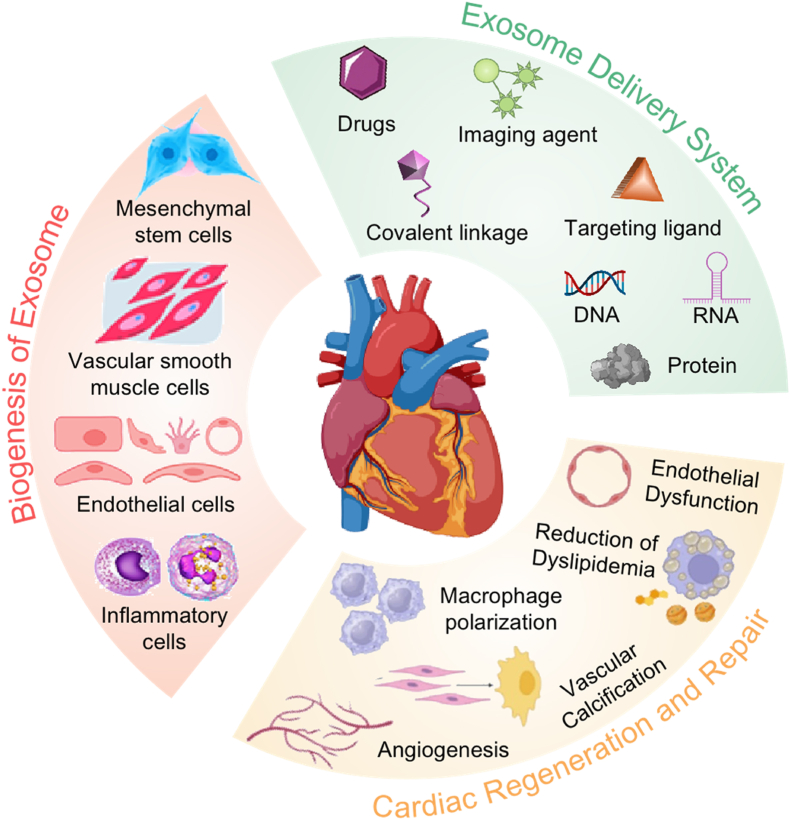
A wheel diagram showing the biogenesis of exosomes and their potential to serve as cargo to deliver a variety of therapeutic agents for cardiac regeneration and repair.

Further clinical studies utilizing novel strategies are necessary to advance the therapeutic application of stem cell therapy for atherosclerosis. This will help in developing a more comprehensive understanding of the potential benefits and limitations of this treatment approach for individuals affected by atherosclerosis. This review aims to provide a comprehensive overview of the therapeutic applications of stem cell-derived exosomes in the context of atherosclerosis origin. We include a detailed explanation of the origin, biogenesis, secretion, and isolation of exosomes. The tailored engineering of exosomes has been summed up to show how the atheroprotective effect can be generated by augmenting exosome therapeutic and targeting capability through drug-loading and surface engineering. This review further elaborates on the pros and cons of different drug-loading strategies as well as how to tailor the exosomes to encapsulate the desired drugs, taking their cytotoxicity and immunogenicity into consideration. Furthermore, a comprehensive discussion has been made on the mechanism of action of drug-loaded exosomes in the treatment of atherosclerosis, which includes the reformation of endothelial dysfunction, dyslipidemia management, polarization of macrophages, inhibition of vascular calcification, and angiogenesis (Scheme 1). In the end, the existing obstacles and possible fixes to improve exosome-based delivery systems for cardiovascular therapies have been provided. In light of this, exosome-based delivery systems represent an intriguing new field in nanomedicine, provided that research in this area continues to improve.

## 2 Atherosclerosis: an overview

The term "atherosclerosis" was coined to denote the lipid substance located within the center of an atherosclerotic plaque or atheroma, which derives from two Greek words: “sclerosis", which denotes stiffening, and “athere”, which means gruel or porridge [15]. Atherosclerotic plaques form due to damage to the inner lining of blood vessels from high pressure and turbulent blood flow. This weakens the vessel barrier by separating cells or opening tight junctions. Endothelial cells (ECs) damage multiple times making them more prone to low-density lipoprotein (LDL) binding [16]. Phospholipid molecules play a significant role in the process of lipid peroxidation, which leads to the oxidation of LDL. One crucial initial step in the early development of atherosclerosis is the entry of LDL particles into the subendothelium (tunica intima), followed by their retention due to ApoB 100 binding to the proteoglycans of the extracellular matrix. As LDL undergoes oxidative changes, ECs express cell adhesion molecules, which attract monocytes and T lymphocytes to the inflamed artery wall [17]. T lymphocytes have the ability to direct the larger macrophages to express genes linked to the development of atheroma and its ultimate instability [18]. The debate continues regarding the roles of CD8^+^ T cells, γδT cells, and other subsets of T helper (TH) cells, including TH2, TH9, TH17, TH22, and follicular helper T (TFH) cells in atherosclerosis. Chemokines and their receptors, such as CCR5, CXCR3, and CXCR6, are responsible for mediating the recruitment of T cells to the atherosclerotic plaque, where natural killer T cells and CD4^+^ TH1 cells are known to play pro-atherogenic roles [19]. Monocytes develop into macrophages, which engulf lipids and transform into foam cells, a characteristic of atherosclerotic lesions. Modified lipoproteins interact with macrophages to initiate pro-inflammatory pathways that enhance further leucocyte recruitment [20]. As the lesion progresses, macrophages and smooth muscle cells (SMCs) may undergo apoptosis. The accumulation of detritus from dying and necrotic cells transforms into the necrotic, lipid-rich core of the atheroma. The impaired efferocytosis in dead cell clearance can also potentially contribute to the development of the necrotic core. Furthermore, the capacity of SMCs to produce interstitial collagen is hindered by T cell mediators such as interferon‐gamma (IFNγ). As a result, the SMCs are unable to effectively maintain and restore the fibrous cap that covers the necrotic core. This can make the lesion more likely to cause clinical events due to increased inflammation, expanding areas of dead tissue, weakening of fibrous caps, and erosion of the endothelial layer [5]. Plaque rupture, erosion, and exposure of necrotic or lipid-rich core components to the systemic circulation induce platelet aggregation, platelet activation, and tissue factor release, all of which are mechanisms by which local thrombosis develops. Thus, the ultimate mechanism through which blood flow is obstructed in atherosclerotic disease is arterial thrombosis [21]. The plaque may also thicken and gather calcium minerals over time. Advanced atheroma also has the potential to invade the artery lumen, obstructing blood flow and resulting in tissue ischemia.

### 2.1 Promoting effects of EVs on atherosclerosis

**Fig. 1:**
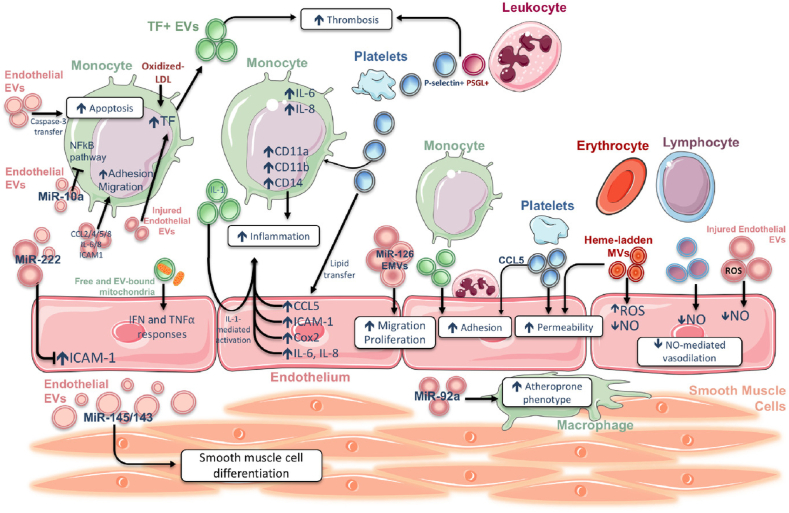
Diagram illustrating the diverse impacts of extracellular vesicles (EVs) produced in vitro, with an emphasis on their function in endothelial function, thrombosis, and inflammation. Adapted with permission from Pierre-Michaël Coly et al. [23].

EVs are released by neutrophils, macrophages, ECs, or vascular smooth muscle cells (VSMCs) that facilitate the transfer of microRNA (miRNA), long non-coding RNA (lncRNA), and proteins in atherosclerosis and contribute to several features of the disease [22]. Varying in composition from protein to nucleic acids, EC-derived EVs are capable of modulating vascular inflammation in distinct ways. They have a multifaceted function; the progression of atherosclerotic lesions can be facilitated or impeded by the payload they transport. While miR-126 stimulates SMC migration and proliferation, the presence of miRNAs (miR-145 and miR-143) in EC-derived exosomes helps preserve VSMC differentiation. Furthermore, endothelial vesicles containing ICAM-1, IL-6/8, and CCL4/5/8 may promote monocyte adhesion and migration. EVs derived from damaged ECs can incite tissue factor (TF) expression and monocyte death, both of which may promote thrombosis. Whereas, miRNA-92a may direct macrophages toward an atheroprone phenotype, and miR-10a in EVs inhibits the NF-κB pathway in monocytes. EVs produced from platelets stimulate adhesion and inflammation in endothelial and monocyte cells. Platelet vesicles and leucocyte-derived vesicles both promote thrombosis. By strengthening leucocyte adherence and triggering the IL-1 pathway in ECs, EVs secreted by monocytes stimulate endothelial inflammation. Moreover, ECs respond to activated monocytes' release of free and EV-embedded mitochondria by producing type I IFN and tumor necrosis factor-alpha (TNFα). Finally, although endothelial, lymphocyte or platelet-derived vesicles block the endothelial NO pathway and reduce vasodilatation, erythrocyte- and platelet-derived vesicles can increase vascular permeability [23] (Fig. 1). In addition, these vesicles are reportedly found in the intimal lesions of both nascent and advanced plaques, indicating that they are involved in both the early and late phases of plaque development. In response to oxidized lipoproteins, ICAM-1 and VCAM-1 are expressed due to pro-inflammatory chemicals in the EC-derived exosomes, exacerbating endothelial barrier dysfunction and impacting the early stages of the disease [24]. EC-derived EVs disrupt the barrier function by destabilizing tight junction proteins through the translocation of c-Src kinase [25]. Moreover, Ya-Ju Chang et al. elucidated the role of miR-92a in CVDs. They demonstrated that the transfer of miR-92a from EC-derived exosomes to macrophages increased the uptake of LDL and the migration of macrophages [26]. MiR-146a-rich macrophage-derived exosomes promote macrophage migration into the vessel wall, accelerating atherosclerosis development [27]. Also, macrophage-derived exosomal miR-185-3p and miR-16-5p, reportedly, exacerbated the development of atherosclerosis in ApoE−/−mice by suppressing Smad7 [28,29]. The migration of SMC and activation of the extracellular signal-regulated kinases (ERK) pathway have been demonstrated to be induced by foam cell-derived EVs, which may worsen lesion progression [30]. By activating ERK5 in vitro, Rap1^+^ EVs derived from platelets also facilitate SMC migration and proliferation [31].

### 2.2 Inhibiting effects of EVs on atherosclerosis

Nevertheless, not all EVs have the potential to cure atherosclerosis. Indeed, certain entities demonstrate a role in preventing the development of atherosclerosis. EVs obtained from bone marrow (BM) dendritic cells (DCs) treated with low-intensity pulsed ultrasonography have a high concentration of miR-16 and miR-21 that effectively suppress TNFα-induced endothelial inflammation by blocking the NF-κB signaling pathway [32,33]. Another study found that the transfer of miR-10a through endothelial EVs suppressed the activation of monocytes by reducing the activity of NF-κB [34]. In terms of functional therapeutic applications, macrophage-derived exosomes can be designed to modulate the immune system and produce substances that aid in the resolution of inflammation [35]. Thus, numerous scientists have studied the functions of specific cell-derived EVs and documented their role in atherosclerotic plaques. The incomplete understanding of the communication between ECs and SMCs in the development of atherosclerosis necessitates further studies.

## 3 Stem cell therapy: the new Frontier

Despite significant progress made in the fields of pharmacological and interventional therapies, surgical procedures, and organ transplantation, there is still a need for additional research into new therapeutic alternative treatments for atherosclerosis. One of the potential treatments for a wide range of heart disorders is stem cell therapy, which has recently gained popularity [36]. Stem cells offer a new and effective method for treating various difficult human diseases due to their ability to prevent inflammation or apoptosis, attract cells, stimulate angiogenesis, and promote differentiation [37]. It has exhibited satisfactory safety profiles and has been shown to be efficacious in the treatment of many conditions, such as acute renal failure, ischemic stroke, and neurodegenerative illness [38,39]. MSC transplantation may also treat ischemia, a clinical outcome of atherosclerosis. MSCs enhance tissue regeneration and angiogenesis in numerous ways. MSC transplantation for ischemia disorders has been hampered by MSC proliferation and differentiation in wounded tissues [11]. Moreover, BM stem cell transplantation reportedly has a beneficial effect on the remodeling of the injured myocardium in patients who have suffered myocardial infarction (MI) [40]. The paracrine factors derived from stem cells play a crucial role in regulating various processes in damaged cardiac tissue, such as ventricular remodeling, inflammation, apoptosis, cardiomyocyte regeneration, and neovascularization [41].

Recent research has demonstrated that the transplantation of MSCs can decrease atherosclerosis in ApoE-deficient mice that were fed a high-fat diet. This finding is relevant to tissue repair and regeneration in CVDs [10]. Stem cells have the ability to regulate lipid levels, suppress inflammation, heal injured tissues, and support hematopoiesis. As a result, stem cells offer a novel approach to treating atherosclerosis due to their beneficial repair and modulation effects on vascular injury and immune response [42]. However, It is reported that MSC-based cell therapy may have a short lifespan and could lead to malignancy, embolism, graft-versus-host disease, and an immune response [43]. Recent studies indicate that the actions of MSCs are mostly mediated by the paracrine production of various soluble substances called exosomes, and not by cell replacement-based tissue repair [44]. Considerable focus has been given to the role of stem cell exosomes, specifically the previously underestimated effectiveness of non-coding RNA found in exosomes on atherosclerosis. This highlights the significance of non-coding RNA in exosomes for regulating atherosclerosis and, optimistically, advancing the development of stem cell therapy for improved clinical use.

## 4 Stem cells-derived exosomes

Exosomes are small EVs that are nanoscale in size and are released by cells. They provide the body with nucleic acids, proteins, lipids, and other bioactive compounds, and they play a part in both the physiological and pathological processes that occur within the body [45]. Exosomes have extensive and distinct advantages when compared to other artificial drug carriers. These advantages stem from their pharmacokinetic and immunologic features, as well as their capability to traverse physiological barriers [46]. Recently, there has been a notable shift towards utilizing stem cell-derived exosomes in the treatment of a range of cardiovascular conditions, encompassing atherosclerosis, stroke, MI, heart failure, peripheral arterial diseases, and pulmonary hypertension [47]. Stem cell-derived exosomes demonstrate similar tissue regeneration and injury repair capabilities as cells themselves. They are also less likely to induce tumorigenicity, graft-versus-host reaction, or immune rejection. Various cell types have been studied so far for the use of stem cell-derived exosomes in a clinical setting. Some cell types, including embryonic stem cells (ESCs), induced pluripotent stem cells (iPSCs), multipotent/unipotent adult stem cell (ASC) lineages like MSCs, cardiac stem cells (CSCs), including cardio sphere-derived cells (CDCs) and CPCs, and endothelial progenitor cells (EPCs), are thought to be safe and effective for generating exosomes because of their prior involvement in cardiac regeneration [48]. ESCs, owning a significant proliferation capacity, provide an intriguing human model to explore the mechanisms governing the proliferation and differentiation of cardiomyocytes [49]. A study found that implanting an ESC in a slow heart-rate pig model made electrical integration and MI regeneration easier [50]. Furthermore, the intramyocardial administration of ESCs-derived exosomes in a murine infarction model promotes cardiac repair and maintains cardiac function, which was attributed to the localization of exosomal miR-294 [51]. As far as iPSCs are concerned, they are easily accessible, remain stable after cryopreservation without losing function, and have the potential to enhance left ventricular systolic functioning and decrease infarct size [[52], [53], [54]]. The iPSCs-derived exosomes introduce cytoprotective properties to cardiomyocytes in vitro and prevent them from myocardial ischemia in vivo; these events are primarily attributed to miRNA-mediated cytoprotection, which inhibits apoptosis. These mechanisms probably relate to their molecular composition, comprising hundreds of miRNAs, proteins, and other growth factors [48,55]. When it comes to CSCs, they reduce the chance of immunological rejection and circumvent moral dilemmas. These cells don't require pre-implantation in vitro differentiation to differentiate into cardiovascular cell types in vivo. These cells seem safe to inject, and prior findings have shown improved myocardial viability, left ventricle ejection fraction, and other cardiac indicators [[56], [57], [58]]. Given their cardiac origins, CPC-derived exosomes might be better suited for cardiomyocyte therapy than stem cells from other sources. The adult heart contains multiple populations of CPCs, including CDCs, which can differentiate into three main cell types: ECs, SMCs, and cardiomyocytes [59]. CDCs are cardiac intrinsic stem cells that express certain profiles of antigens, including CD45^+^ and CD105^+^, which contribute towards the functional recovery of various forms of heart failure [60]. CDC-derived exosomes were shown to convert dermal fibroblasts into active cells reducing MI size and improving cardiac function [61]; whereas, their regenerative and functional role appears in cells exosomal miR-146a following direct transplantation to a bruised heart [62]. C-kit^+^ stem cells, given the fact that C-kit^+^ cells have cardiac differentiation capacity, are considered to be among the pioneering cells in research and have already drawn interest in experimental in vitro models [63,64], though their function in myocardium regeneration remains vague [65]. The age of the donor may be one of the most significant variables influencing the effectiveness of CPCs. Numerous research comparing adults and newborns have demonstrated that neonatal C-kit^+^ cells proliferate more quickly in vitro than adult C-kit^+^ cells, which results in better cardiac repair following coronary artery ligation in rat models [66]. Furthermore, following pre-stimulation with ESC-derived exosomes, CPCs showed an up-regulation of certain EC and cardiomyocyte gene expression. Additionally, iPSC- and ESC-derived exosomes enhanced tube formation and proliferation in CPCs, although in vitro apoptosis was decreased in both cardiomyocytes and CPCs. The administration of CPCs stimulated with ESC-derived exosomes decreased infarct size and improved cardiac performance, whereas it also raised vascular density and proliferation. Exosomes include a variety of identified miRNAs that may be responsible for re-inducing and promoting the growth of CPCs in vitro [51]. The regenerating potential of CPC-derived exosomes has been the subject of some research. For instance, under various circumstances, mice/rat-CPC-derived exosomes promote the tube formation of ECs, decrease cardiomyocyte apoptosis, impede fibrosis, and enhance cardiac function [67]. Human-CDC-derived exosomes may also enhance cardiac performance and vessel density, inhibit fibrosis, and decrease the size of the infarcted area within 7–30 days following MI [62].

EPCs can be easily extracted from a variety of sources with minute modifications. They release angiogenic factors and are essential for in vivo cardiovascular regeneration. In the case of coronary atherosclerosis, they take part in the restoration of the heart and blood vessels. Circulating EPCs are drawn to injury sites after tissue damage, facilitating vascular repair [58,[68], [69], [70]]. Likewise, MSCs impart simple isolation and amplification with minimal immunogenicity and have demonstrated the ability to vascularize and improve heart function [71]. MSCs can be derived from various tissues, notably BM, adipose tissue, amniotic fluid, and umbilical cord blood (UCB), among others [72,73]. One characteristic that sets them apart is their propensity to differentiate into pertinent cells such as osteogenic, adipogenic, chondrogenic, neurogenic, etc. In cardiac regeneration, MSC-derived exosomes are gaining a lot of interest. These exosomes secreted cytokines and induced the differentiation of diverse phenotypes in vitro with commendable efficacy [74,75]. These exosomes are known to significantly impact various aspects of human umbilical vein ECs. They enhance the formation of lumens, hinder the proliferation of these cells in a laboratory setting, decrease the area affected by atherosclerosis, and help maintain the proper functioning of the heart during both the systolic and diastolic phases. As a result, they effectively increase the density of capillaries and improve blood flow at the site of ischemia in atherosclerosis [76]. In a rat model of MI, miRNAs in exosomes derived from human endometrium-derived MSCs demonstrated cardioprotective properties, with miR-21 being credited with the cardioprotective effect [77]. UCB-MSCs-derived exosomes were proven to boost angiogenesis and MI tissue regeneration under hypoxic conditions; the cardioprotective impact was attributed to miR-210 [78]. Therefore, exosomes derived from other adult stem cells may also garner significant interest as a potential substitute for cell-based therapy in the treatment of CVDs [48]. It is important to ponder that exosomes derived from different cell types show significant potential in restoring cell damage with multiple programmed deaths.

## 5 Biogenesis of exosomes

The most recent working guidelines refer to these nanoparticles as EVs, even though they have been studied under several names over the past few decades, including exosomes, ectosomes, and oncosomes [79]. An intracellular sorting/trafficking mechanism was identified through an extensive study on EVs and then used to characterize the release of exosomes that occur when multivesicular bodies (MVBs) fuse with the apical plasma membrane [80]. This paved the way for several decades of EV study, some of which focused on important EV features like flippase activities, lateral diffusion in vesicle membranes, and essential parts like Rab, ARF, and tetraspanins. The composition of EVs can differ in response to specific stress circumstances, such as altered iron balance [81]. The five cell types utilized to generate EVs following Good Manufacturing Practice (GMP) are human CPCs, BM-MSCs, adipose-derived stem cells (ADSCs), monocyte-derived DCs, and HEK293 cells [82].

**Fig. 2:**
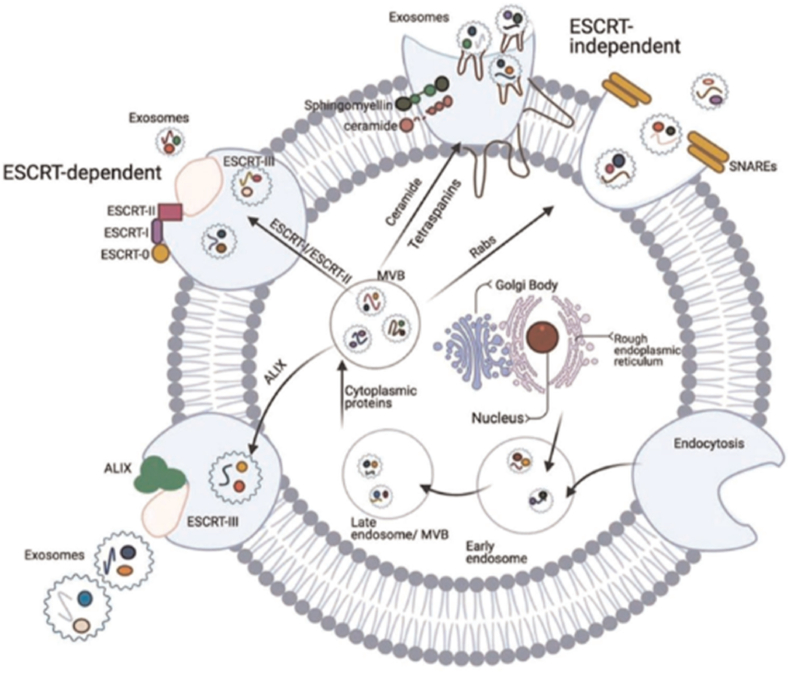
Biogenesis of an Exosome. ESCRT-dependent and Independent Pathways. Adapted with permission from Ghadi N. Alzhrani et al. [83].

The endosomal sorting complex required for transport (ESCRT), which is made up of a combination of proteins and carbohydrates, is necessary for several stages of exosome biogenesis via MVB development (cargo assembling, bud growth, and vesicle fission). It is a distinct process consisting of approximately 30 proteins that form four complexes (ESCRT-0, -I, -II, and -III) (Fig. 2). This mechanism regulates the formation of intraluminal vesicles (ILVs). Furthermore, the formation of exosomes involves two important proteins, namely TSG101 and ALIX [83]. Early-acting ESCRT-0, -I, and -II assemble in the cytoplasm, where ESCRT-III acts exclusively on membranes and is late-acting [84]. ESCRT-0 and -I bring cargo and ESCRT-II. By causing membrane distortion, ESCRT-I and -II aid in the stabilization of the membrane neck. ESCRT-II then attracts ESCRT-III, which drives vesicle neck fission and brings in the Vps4 complex to enhance ILV budding. Additionally, this stage is crucial for recycling the ESCRT pathway for use in later sorting rounds [85].

Contrary to this, exosomal biogenesis can also be achieved independently of the ESCRT pathway, primarily comprising lipids, heat shock proteins, and tetraspanins [86]. It was proven by ILV biogenesis inside the lumen of multivesicular endosomes (MVEs) in ESCRT-deficient cells [87]; the earliest ESCRT-independent machinery that was found to rely on sphingolipid ceramide [88]. CD63, one of the tetraspanins, has been shown to enable the ESCRT-independent sorting of melanosomal protein into ILVs during melanogenesis [89]. Another research documented the contribution of chaperone HSC70 in the biogenesis of exosomes [90]. Another latest research found that epidermal growth factor phosphorylated RAB31 plays a critical role in the biogenesis of exosomes by promoting ILV production and inhibiting MVE depletion [91]. In addition, the regulation of MVB transport is influenced by various factors, including the Rab GTPase family, cytoskeleton, molecular motors, and membrane fusion devices such as the SNARE complex [92,93]. The miRNA retains its functional integrity in the receiving cells. Hence, they are well-suited for EV-based therapy. Exosomes enrich certain miRNAs while hardly containing others, suggesting the existence of highly selective RNA sorting mechanisms [94]. Argonaute 2 (Ago2), a component of the RNA-induced silencing complex (RISC), can control the inclusion of specific miRNAs, such as let-7a, miR-100, and miR-320a, into exosomes. Moreover, Vps4A, a type of protein found in cell membranes, was involved in the process of organizing miRNAs into exosomes. When the activity of Vps4A was blocked, the levels of exosomal miR-92a and miR-150 were reduced [95,96]. Another perspective holds that the mechanisms are not distinct; they may impact or function in conjunction [97]. Certainly, exosome biogenesis is regulated by extremely complex systems. Under many physiological and pathological events, these pathways are tightly controlled and influenced by both internal and external factors [98].

## 6 Secretion and isolation of exosomes

**Fig. 3:**
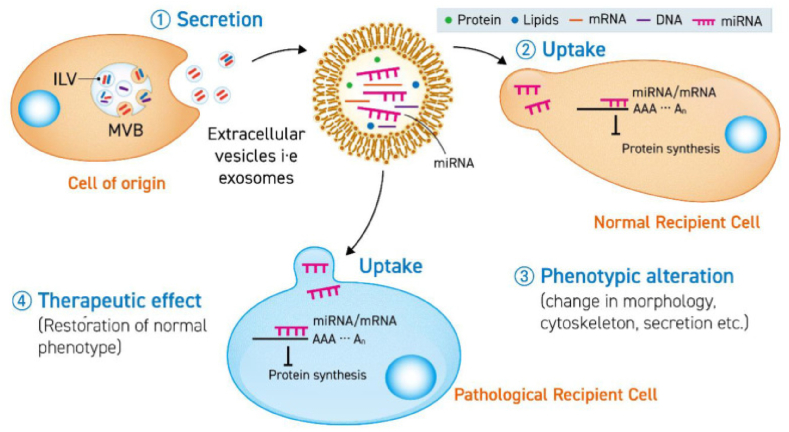
The released extracellular vesicle (EV) containing miRNA is internalized by either a healthy or diseased cell, leading to a phenotypic alteration or therapeutic outcome through the suppression of mRNA translation in the receiving cells. ILV is an abbreviation for intraluminal vesicles, while MVB is an acronym for multivesicular bodies. Adapted with permission from Javaria Munir et al. [101].

Similar to the multiple mechanisms proposed for exosome synthesis, numerous mechanisms have been proposed for exosome release. Numerous Rab GTPases, including RAB11 and RAB35, or RAB27A and RAB27B, are known to be important [99]. Proteins play a crucial role in regulating the fusion of MVBs with the outer membrane to the plasma membrane, leading to the release of exosomes (Fig. 3). There have been descriptions of five distinct machineries up to this point. One of these machineries involves RAB11 and RAB35, which aid in the fusion of MVBs to the plasma membrane. This fusion process results in the release of exosomes that contain proteolipoprotein (PLP), Wnt, flotillin, and transferrin receptor (TfR) [100]. Furthermore, exosomes can also be released from the plasma membrane by budding on their own without the assistance of Rab GTPases.

EVs can be acquired from various sources, such as bodily fluids and conditioned media. Before EV isolation for further uses, blood serum can be kept at a temperature of −80 °C at which they can be stored for up to 30 years [102]. Various methods are used to separate exosomes, taking into account their physical and chemical characteristics such as density, size, and surface components. These methods include ultracentrifugation (UC), size-based isolation techniques, charge neutralization-based polymer precipitation, immunoaffinity, and microfluidic techniques [103]. Currently, advanced exosome separation methods like immunoaffinity, chromatography, and polymer precipitation have been developed [104]. The classic polymer-based precipitation approach has been improved to overcome the persistent challenge of hydrophobic protein interference in isolating urine exosomes [105]. To ascertain whether the identified components are exosomes, multiple characterization indices are required because exosome characterization is distinct and identical. In recent decades, several exosome biomarkers, including TSG101, CD81, CD9, CD63, CD37, CD82, CHMP2A, ALIX, RAB11B, CHMP4B, RAB11A, and RAB5, have been studied to isolate exosomes using immunoaffinity techniques. Based on 50 years of accumulated experience, it has been found that there are no 100 % specific markers for normal cells, stem cells, or tumor cells. Even well-known exosome markers such as CD63 and CD81 display expression on different subcellular organs or microvesicles [106,107].

## 7 Exosome delivery systems for the treatment of atherosclerosis

A well-chosen route of administration within the drug delivery system may improve its therapeutic impact. Exosome efficacy and biodistribution are greatly affected by the mode of administration [108,109]. There are several ways to administer the exosome delivery system; the most popular ones include intravenous injections [110], subcutaneous injections [111], intraperitoneal injections [112], nasal administration [113], and oral administration [114]. Intravenous injection is the most popular method of administration when using exosomes as drug delivery agents [115].

### 7.1 Techniques for loading biological ‘cargo’ into exosomes

Loading biological cargo into exosomes offers a viable approach for significantly increasing therapeutic efficacy by maintaining its integrity in the in vivo environment and improving its biodistribution [116]. Several methods have been developed for effective loading into exosomes, providing opportunities for establishing cutting-edge therapeutic approaches.

**Fig. 4:**
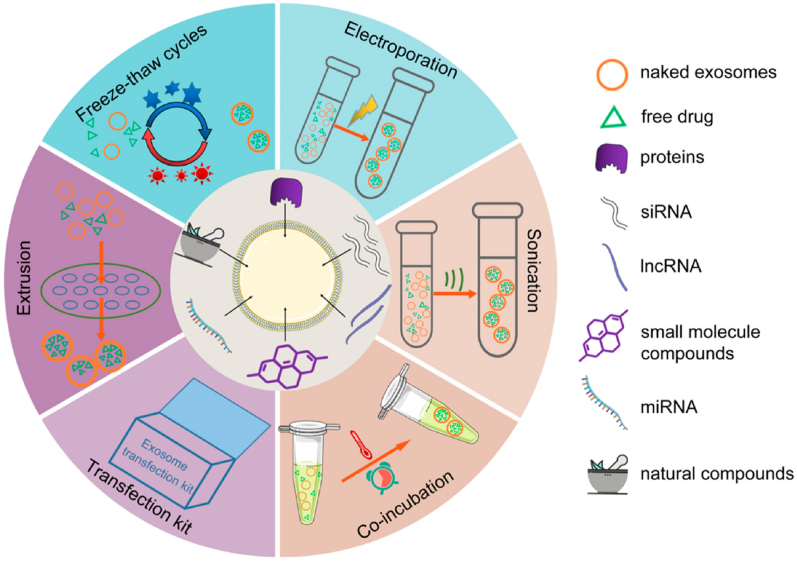
Post-secretory methods for drug-loading into exosomes include freeze-thaw, electroporation, sonication, co-incubation, extrusion, and so on. Pre-secretory drug-loading methods into exosomes include transfection. Adapted with permission from Sun et al. [115].

Drug delivery via exosome is categorized according to whether the therapeutic agent is directly placed onto the exosome: pre-secretory (indirect drug-loading) and post-secretory (direct drug-loading) [116,117]. Pre-secretory method involves drug-loading into donor cells before exosome isolation, which enables the drugs to be encapsulated within exosomes during their natural biogenesis. Pre-secretory loading modifies the donor cells by either transfecting the target gene to produce tailored exosomes or co-incubating donor cells with the payload [118,119]. These altered cells have been engineered to generate and release exosomes that carry certain payloads, including proteins, nucleic acids, and drugs. Loading proteins, siRNA, miRNA, and other high molecular weight drugs is beneficial among them [120,121]. The pre-loading technique preserves membrane integrity while enabling the continual synthesis of exosomes loaded with payload. Although pre-secretory drug delivery is easy, the primary constraint is the poor drug-loading efficacy. Furthermore, the approach may cause toxicity from transfection agents and can alter donor cell gene expression, thus interfering with the normal physiological functions of membrane proteins [45,122]. There are two different approaches under post-secretory loading: active loading and passive loading [121]. While active loading employs several methods, including electroporation, sonication, and extrusion, for drug-loading into the exosomes, passive loading depends on the physicochemical characteristics of the drug to inertly diffuse into exosomes [122,123]. The post-secretory approach is more efficient than pre-secretory because it gives more control over the loading efficacy of the ultimate product [124]. Hence, post-secretory drug delivery techniques are frequently employed (Fig. 4) [[125], [126], [127]].

Among the aforementioned loading techniques into exosomes, the most frequently employed methods are electroporation and sonication. By using either technique, the lipid bilayer is temporarily perforated, permitting the payload to diffuse into the exosome. Sonication uses an ultrasonic probe to apply mechanical force to induce cavitations that physically disrupt the exosome membrane, permitting cargo to diffuse into exosomes [128]. Scientists have reported good loading efficiency into exosomes using this method. For example, by using sonication, Yerneni et al. successfully loaded albumin and curcumin into exosome carriers with great stability and anti-inflammatory properties [129]. On the other hand, electroporation is a simple and effective method for cargo-loading into exosomes that preserves the original characteristics of the payload and delivers a higher loading efficiency as compared with other methods [130]. Using an electric field, electroporation lowers the energy threshold needed for the membrane pore formation by creating a voltage drop across the membrane. This raises the frequency of pore formation and makes it easier for loading moieties to diffuse into the exosome [131]. Several reports have validated the practicality of a variety of payloads electroporated into exosomes for atherosclerosis therapy. MSC-based exosomes have been recognized for the treatment of atherosclerosis. For instance, Gao et al. treated oxidized LDL (ox-LDL)-induced eosinophils with human umbilical cord MSC-derived exosomes. They found that there was a decrease in the amount of inflammatory cytokines, an inhibition of cell migration, and an increase in cell apoptosis [132]. However, the electroporation settings may affect the loading efficiency. Lennaárd et al., analytically assessed several electroporation optimization parameters, including the amount of EVs, drug-to-vesicle ratio, buffer solution, pulse capacitance, and field strength. They demonstrated that loading efficacy can be considerably improved under appropriate electroporation conditions [133]. Even while sonication has the edge of continual drug release and improved drug-loading efficacy, exosomes may be negatively impacted by sonication in particular. It may result in the loss of cargo and membrane reformation, both of which are crucial for the therapeutic benefits of exosomes [134,135]. Therefore, more investigation is needed to ascertain how sonication impacts the exosome structure, intrinsic cargo, and lipid and protein content of membranes. Furthermore, the loading method may potentially result in morphological alterations and exosome aggregation [136]; the impact of these modifications on treatment efficacy has not yet been determined but has to be looked into in further research. Nevertheless, sonication has been successfully used in loading cargo into preclinical exosome formulations and has shown tremendous potential for optimizing drug delivery which makes it a preferred technique for creating innovative exosome drug delivery systems. However, the active loading method has drawbacks of its own. For example, it has been demonstrated that alterations to liposome size, a comparable nanoparticle platform to exosomes, have a notable impact on biodistribution, circulation time, and recognition by reticular endothelial systems [137]*.* Regardless of the approach chosen for practical implementation, it is crucial to evaluate several parameters, including drug characteristics, drug loading efficacy, and damage to exosome membranes due to physical force, pH fluctuation, and surfactants. Choosing the most optimal strategy is therefore crucial, especially in light of the many technologies that make it possible to customize the exosomes' payload to fulfill certain therapeutic requirements.

### 7.2 Surface engineering of exosomes for targeted delivery

**Fig. 5:**
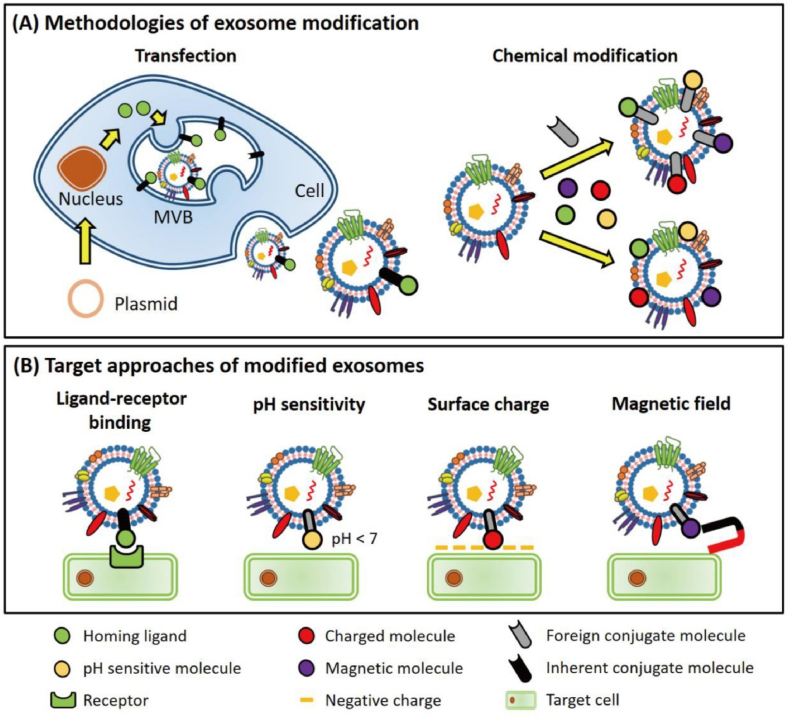
Approaches for designing targeted exosomes. To obtain targeting capacity, native cargos are assembled on the exosomal membrane via transfection or chemical modification (A). Native cargo-coated exosomes are directed to target cells via ligand-receptor binding, pH gradient, electric magnetism, or magnetic field (B). Adapted with permission from Fu et al. [148].

Apart from the fact that there is a lack of a standard methodology for incorporating desired payload into exosomes, there is yet another obstacle to overcome before employing an exosome-based delivery system in clinical settings and scientific exploration. Various sources suggest that distinct cell types could possess varying abilities to capture exosomes [138,139]. It is probably because different processes that are employed in exosomes, such as phagocytosis, micropinocytosis, lipid raft-mediated endocytosis, clathrin-mediated endocytosis, and caveolin-dependent endocytosis, have varying exosome internalization efficiencies [140]. Nonetheless, a variety of data indicate that naturally occurring exosomes diffuse freely across extracellular spaces and biofluid before internalizing at random into target cells. For instance, observing fluorescent dye/probe-labeled exosomes in the liver, spleen, kidney, pancreas, and other organs can reveal patterns of the unregulated biodistribution of exosomes [108,109]. Hence, to deliver intended payloads to targeted cells or tissues, methods that provide naturally occurring exosomes with targeting ability are also required (Fig. 5).

Chemical or transfection-mediated surface modification approaches are commonly employed (Fig. 5A). Chemical surface functionalization usually involves click chemistry and amphiphile insertion methods to display targeting moieties [[141], [142], [143], [144], [145]], while the transfection-mediated approach alters the exosome via transmembrane fusion protein to exhibit targeting agents [146]. Lysosome-associated membrane protein 2B (LAMP-2B) is the most often employed transmembrane protein with its exposed N-terminus on the exosome surface, which may be utilized to conjugate targeting moieties [[147], [148], [149]]. Intrinsically, this approach can accept a wide range of peptides and ligands that target monocytes and macrophages implicated in atherosclerosis and display them on the exosome surface (Fig. 5B) [[150], [151], [152], [153], [154], [155]]. In contrast, the click chemistry approach modifies the amine groups of transmembrane exosomal proteins with alkyne groups. These groups are subsequently conjugated to azide-containing targeting ligands by copper-catalyzed azide-alkyne cycloaddition "click" reactions [143]. However, conjugation is non-specific and can amend different transmembrane proteins, albeit being comparatively more rapid [145,147]. In addition to click chemistry, amphiphile insertion is a relatively easy way to introduce targeting moieties and modify the exosome membrane via incubation and sonication. However, it is yet unknown how sonication affects the exosome membrane's integrity [135]. Nevertheless, these approaches provide a toolset for designing exosomes to transport atheroprotective cargo and to improve their targeting capacity.

### 7.3 Delivery of small-molecule drugs via exosomes

**Fig. 6:**
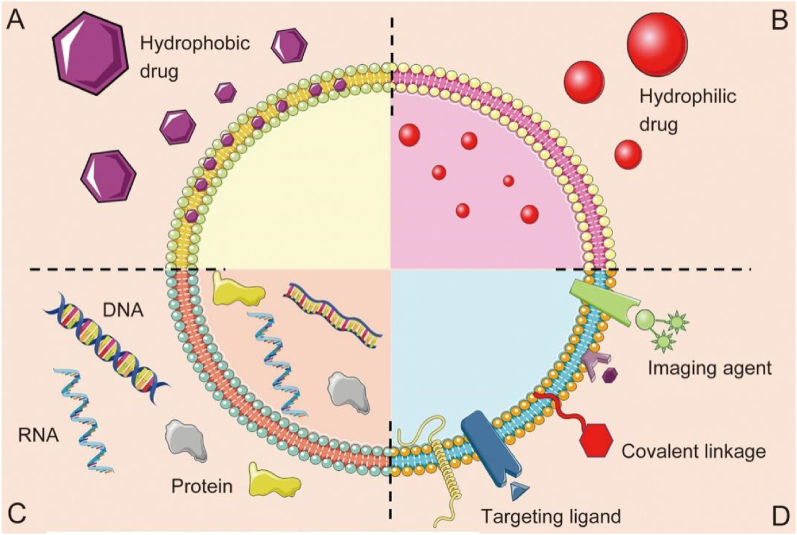
Exosomes Delivery Systems. Exosomes consist of a lipid bilayer, which encloses an aqueous core. Both the lipid bilayer and the aqueous core can integrate hydrophobic (A) or hydrophilic moieties (B), respectively. Exosomes can incorporate nucleic acids and protein (C). Exosomes can be designed to improve targeting ability via surface modification by conjugating theranostic or imaging agents, specific targeting ligands, and covalent linkage to the exosome membrane (D). Adapted with permission from Luan et al. [157].

For encapsulating small-molecule drugs, the active loading method is frequently used. This method purposely disrupts the exosome membrane by co-incubation, freeze-thaw, electroporation, and ultrasonic treatment to permit drugs to diffuse effectively inside the exosome (Fig. 4) [[156], [157], [158]]. However, the drug-loading into exosomes is determined by the characteristics of the drugs to be encapsulated (such as size, solubility, stability, effective dosage, etc.) and the degree to which the method affects the structure and content of exosomes. For example, hydrophobic drugs can load inside the hydrophobic portion of the lipid bilayer without totally impairing the integrity of the membrane (Fig. 6A). However, hydrophilic drugs need active loading methods that significantly disrupt the exosome membrane to facilitate drug penetration into the hydrophilic exosome core (Fig. 6B) [[159], [160], [161]]. After administration, exosomes typically result in increased drug distribution in the targeted area and better stabilization and prolongation of blood circulation time for small molecules, ultimately enhancing the efficacy of the small-molecule drugs. For instance, curcumin was loaded into exosomes via the co-incubation method, which has been demonstrated to improve curcumin stability in vitro and increase blood levels in vivo. Thus, the curcumin levels increase in target cells, wherein inflammatory cytokines, including IL-6 and TNF-α, are significantly suppressed in a lipopolysaccharide-induced septic shock mice model [159]. The majority of researchers, however, encapsulate anticancer drugs into exosomes to treat tumors specifically. Further investigation is required to examine the connection between exosomes and atherosclerotic drugs.

### 7.4 Delivery of therapeutic RNAs via exosomes

In the context of biologics, nucleic acids can be encapsulated into exosomes (Fig. 6C) using active loading or cell transfection methods (Fig. 4). In CVDs, MSC-derived exosomes can be used to deliver RNA to the injury site, which can reduce cardiac fibrosis, improve cardiac recovery, and promote angiogenesis. In a study, miR-132 was electroporated into MSC-derived exosomes to examine its impact on angiogenesis; wherein miR-132 targeted RAS p21 protein activator-1 and enhanced tube formation of ECs in vitro. In the MI mice model, MSC-derived exosomes ameliorated angiogenesis at the site of infarction and promoted cardiac recovery [162]. Correspondingly, MSC-derived exosomes expressing the let 7 and miR-512-3p families are also being used as therapeutic agents to treat atherosclerosis, proving the immense potential of MSC-derived exosomes for the treatment of atherosclerosis [163,164]. Another study transfected the miR-126 with MSC-derived exosomes and evidenced its delivery to ECs. This improved angiogenesis and survival in ECs impaired by hypoxia/reoxygenation by stimulating the PI3K/Akt/eNOS pathway, inhibiting cleaved caspase-3 expression, and activating the expression of pro-angiogenic factors [165]. Apart from activating angiogenesis, RNA incorporated into MSC-derived exosomes can prevent cardiac fibrosis in MI. To investigate the effect on MI, another study transfected the miR-19a/19b into MSC-derived exosomes and delivered them into the area of MI during MSC transplantation. In a mouse model of MI, MSC-derived exosomes reduced myocardial fibrosis and inhibited the apoptosis of cardiac HL-1 cells. However, reducing cardiac fibrosis only lessened its negative effects; it did not impede fibrosis [166]. Through the innovative transplantation of MSCs and MSC-derived exosomes into the infarcted region, cardiac fibrosis can be reduced with improved cardiac function. Although this study offered a novel “amalgam” developed in the area of myocardial protection, more systematic research is required to confirm the biosafety of this approach. Moreover, studies have shown miRNAs play a crucial role as an exosome mediator. However, additional research is required on other miRNA mediators, for instance, miR-146a. According to one study, in a mouse model of MI, delivering miR-146a reduces the size of the infarcted area and enhances cardiac performance; in contrast, no improvement was seen without miR-146a [167].

### 7.5 Delivery of therapeutic proteins via exosomes

One popular technique for encapsulating proteins is to transfect cells with plasmids encoding the desired proteins (Fig. 4) [125,147,168]. Currently, overexpressing the protein is primarily used to load it into exosomes. To investigate the potential for targeted delivery of exosome-loaded proteins, a study transfected overexpressed protein kinase B (Akt) with MSCs derived from the human umbilical cord and explored whether exosomes released from Akt-overexpressing MSCs had a positive impact on angiogenesis and cardiac protection. According to the findings, it speeds up migration and proliferation of ECs, and angiogenesis in vitro and considerably boosts cardiac function in vivo. The scientists noted that this might be connected to the up-regulation of platelet-derived growth factor D expression by Akt-MSC-derived exosomes [73]. However, it is still uncertain if modifying the protein content will have an impact on other cellular processes. Additionally, research has shown that exosomes are more likely to function with proteins instead of RNA [147]. Thus, more research is required to explore the RNA and protein-based mechanisms and validate exosomes’ viability as a protein delivery vehicle.

A different strategy for targeted delivery via exosomes is to ingeniously anchor the "payload" on the exosomal surface rather than encapsulating it. Thus, not only can exosomes carry the payload inside them (Fig. 6A–C), but they are also tailored to improve their targeting ability by altering their surfaces (Fig. 6D). Exosomes' selectivity as “payload” carriers makes it possible to treat a wide range of inflammatory disorders while avoiding negative effects from off-targets. In particular, tailoring them to contain certain targeted moieties and trigger specific activators through their contents, shows potential for use in the treatment of atherosclerosis [169]. These findings heighten hopes for a more focused anti-inflammatory impact from exosomes and eventually manage the remission of atherosclerosis. Inclusively, the developments in drug-loading techniques are encouraging for targeted drug delivery and may have implications for future medicinal applications.

## 8 Mechanism of action

**Fig. 7:**
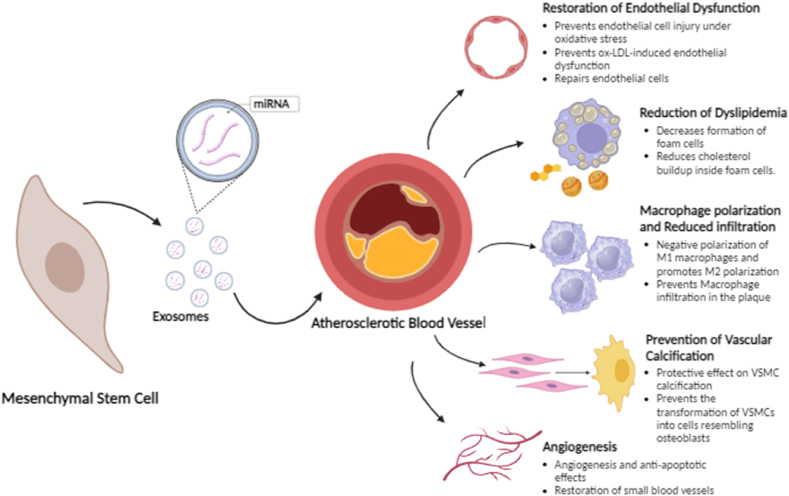
Therapeutic mechanisms of exosomes in atherosclerosis management.

MSCs, with their capacity to induce immunomodulation and regenerate tissue, have become a popular therapeutic option for treating a number of illnesses, including atherosclerosis. Mounting evidence confirms their preventive function at all stages of atherosclerosis. Stem cell-derived exosomes have the potential to treat atherosclerosis by restoring endothelial functions, reducing oxidative stress, decreasing dyslipidemia, and mitigating macrophage infiltration by decreasing the production of inflammatory mediators and angiogenesis (Table 1) (Fig. 7).

**Table 1:** Mechanism of Action of Stem-cell derived Exosomes for the Treatment of Atherosclerosis.

Source	Regulatory Molecules	Pathways	Function	Reference
MSCs-derived Exosomes	miR-342-5p	PPP1R12B	Prevent EC injury under oxidative stress	Xing X et al. [171]
MSCs-derived Exosomes	miR-512-3p	Keap1	Prevent ox-LDL-induced endothelial dysfunction	Chen S et al. [163]
MSCs-derived Exosomes	–	soluble forms-like tyrosine kinase-1 (sFlt-1)	Prevent endothelial dysfunction and promote angiogenesis	Chang X et al. [172]
EPCs-derived Exosomes	miR-223, miR-21, miR-126, miR-146a		Repair ECs	Alexandru N et al. [173]
Platelet membrane-coated exosome-mimetic nanovesicle		ABCA1 and ABCG1 upregulation	Reduced cholesterol buildup inside foam cells.	Jiang Y et al. [175]
MSCs-derived Exosomes	miR-221-3p, miR-34a-5p and miR-16-5p let-7a-5p, miR-143-3p	upregulation of UCP2 and MnSOD genes PI3K and pAKT4 downregulation	Restore the antioxidant states of cardiac mitochondria	El-Derany MO et al. [177]
MSCs-derived Exosomes	miR-let7	HMGA2/NF-κB	Infiltration and polarization of M2 macrophage	Li J et al. [164]
MSCs-derived Exosomes	miR-182	inhibiting the TLR4/NF-κB pathway activating the PI3K/AKT pathway	Negative polarization of M1 macrophages and promotes M2 polarization	Zhao J et al. [181]
MSCs-derived Exosomes	miR-let7	IGF2BP1/PTEN	Prevent Macrophage infiltration in the plaque	Li Q et al. [180]
MSCs-derived Exosomes	miR-21a-5p	KLF6 and ERK1/2	Promote M2 polarization and prevent macrophage infiltration	Ma J et al. [179]
MSCs-derived Exosomes	miR-145	JAM-A	Inhibition of cellular migration at the site of lesion	Yang W et al. [182]
MSCs-derived Exosomes	miR-148a-3p	expression of CNTN4	Decrease IL-6 and TNF-α in THP-1 macrophages stimulated by ox-LDL	Wang K et al. [184]
BM-MSCs-derived exosomes	lncRNA	NONHSAT 084969.2/nuclear factor-κB pathway	Prevent vascular calcification	Liu Y et al. [187]
BM-MSCs-derived exosomes	miR-15a/15b/16	Inhibit NFATc3 OCN down-regulation	Prevent vascular calcification	Luo F et al. [188]
MSCs-derived Exosomes	miR-146a	Src pathway	Promote Angiogenesis	Xiao X et al. [191]
CPC-derived Exosomes	pre-fibrosis gene↑	TGF-β ↑	Anti-apoptotic and angiogenic effects	Teng X et al. [193]
MSCs-derived Exosomes		SDF1	Restoration of small blood vessels	Gong XH et al. [194]

### 8.1 Restoration of endothelial dysfunction

Endothelial dysfunction is regarded as the initial alteration in the development of atherosclerosis, occurring before the detection of atherosclerotic plaque through investigations [170]. MSCs from adipose tissue and microRNA-342-5p carried by exosomes can prevent the programmed cell death of ECs in blood vessels triggered by oxidative stress in atherosclerosis [171]. It is also reported that MSCs protect ECs from ox-LDL damage by secreting interleukin-8 (IL8) and macrophage inflammatory protein-2 (MIP-2). IL8 prevents neutrophil adhesion and promotes EC survival, proliferation, and angiogenesis. MSCs also enhance EC functioning through p38 MAPK activation. The MSCs themselves or the substances they secrete can potentially be used to cure atherosclerosis in patients [10]. Chang et al. demonstrate that human umbilical cord MSC-derived exosomes can reverse sFlt-1 (Soluble fms-like tyrosine kinase-1) induced preeclampsia birth outcomes in vivo [172]. These vesicles aid human umbilical vein ECs sFlt-1-impaired endothelial dysfunction. MSCs derived exosomal miR-512-3p regulates Keap1 (Keleh-like ECH-associated protein-1) to reduce ox-LDL-induced vascular ECs dysfunction. Exosomes enriched in miR-512-3p from MSCs also significantly reduced ox-LDL-mediated EC damage and proliferation [163]. Administering allogenic EVs from healthy sources might mitigate the harmful effects caused by a high-fat diet by transferring biologically active miR-10a, miR-21, miR-126, and miR-146a to circulating EPCs. This transfer facilitates the repair of blood vessels and helps in the treatment of atherosclerosis [173].

### 8.2 Reduction of dyslipidemia

Dyslipidemia is one of the important hallmarks of atherosclerosis. To expedite the clinical implementation of MSCs, researchers have developed numerous animal models of atherosclerosis and conducted extensive studies. Studies have discovered that BM-MSCs have the ability to decrease irregularities in blood lipids [42]. Zhang showed that by decreasing the expression of SR-A1 and CD36 and increasing the expression of ABCA1, human GMSCs significantly decreased the formation of foam cells and accumulation of lipids in macrophages [174]. Additional in vitro studies also demonstrated that platelet membrane-coated exosome-mimetic nanovesicles stimulated the removal of cholesterol and reduced the buildup of cholesterol inside cells by increasing the expression of the essential cholesterol transporters ABCA1 and ABCG1 [175]. Also, the delivery of exosomes derived from ADSCs or MSCs, in the mice resulted in a notable decrease in the concentrations of total cholesterol, LDL-cholesterol, and triglycerides in the plasma [176]. The MSC-derived exosomes were able to restore the antioxidant states of cardiac mitochondria in non-alcoholic steatohepatitis. Additionally, they derange both Parkin-dependent and Parkin-independent mitophagy processes through the downregulation of PI3K and pAKT pathway [177].

### 8.3 Macrophage polarization and reduced infiltration

Macrophages are a diverse population that can exhibit various phenotypes and functions in response to changes in the surrounding microenvironment. Macrophages can be polarized into multiple functional phenotypes, depending on their activation status. These phenotypes include classically activated macrophages (M1) and alternatively activated macrophages (M2) [178]. In atherosclerotic plaque tissues of mice with atherosclerosis, exosomes derived from MSCs decisively decreased the proportion of M1 macrophages while markedly increasing the proportion of M2 macrophages. Macrophages polarized M2 effectively penetrate atherosclerotic plaques and inhibit the local inflammatory response [179]. Another study reports that exosomes ameliorated atherosclerosis in ApoE−/− mice and promoted M2 macrophage polarization in the plaque via the miR-let7/HMGA2/NF-κB pathway [164]. Skin derived-MSCs exert a beneficial influence on macrophage response by increasing prostaglandin E2 release, promoting IL-10 production and release as an anti-inflammatory cytokine, and reducing the production of tumor necrosis factor-α, a pro-inflammatory cytokine [180]. When enclosed within exosomes formed from MSCs, miR-182 specifically targets TLR4, thereby inhibiting the TLR4/NF-κB pathway and activating the PI3K/AKT pathway in the context of cardiac ischemia/reperfusion (I/R) injury. Consequently, this also leads to the negative polarization of M1 macrophages and promotes the polarization of M2 macrophages in the heart [181].

Furthermore, they decisively inhibited macrophage infiltration into the plaque via the miR-let7/IGF2BP1/PTEN pathway [180]. Targeting the KLF6 and ERK1/2 signaling pathways can also limit the invasion of macrophages at the site of the lesion, thus mitigating the development of atherosclerosis [179]. Moreover, the administration of exosomes enriched with miR-145 resulted in the suppression of JAM-A, leading to the inhibition of cellular migration in vitro, and a decrease in atherosclerotic plaque formation in living organisms [182]. Yoshimasa et al. also report that humoral factors, such as hepatocyte growth factor (HGF) and EVs derived from MSCs, show potential as therapeutic agents for mitigating the remaining risks associated with atherosclerosis [183]. Moreover, exosomes containing miR-148a-3p also greatly suppressed the expression of CNTN4, cell death, and the levels of IL-6 and TNF-α in THP-1 macrophages stimulated by ox-LDL [184]. Therefore, exosomes produced from MSCs show potential as a viable treatment for atherosclerosis.

### 8.4 Prevention of vascular calcification

The calcification of the artery wall is an active process principally caused by the transformation of VSMCs into cells resembling osteoblasts [185]. MSC transplantation was found to have a significant protective effect on VSMC calcification through a paracrine mechanism. Research conducted in vitro has demonstrated that downregulating Wnt signaling pathways and controlling bone-related indicators to prevent the osteogenic differentiation of VSMCs may have an impact on the capacity of MCSs to regulate the immune system and produce paracrine effects [186]. BM-derived exosomes were discovered to prevent vascular calcification caused by elevated levels of phosphorus in VSMCs in an in vitro setting. This phenomenon appears to be facilitated by the suppression of genes associated with calcification, including RUNX2, BGLAP, and BMP2, as well as the prevention of calcium buildup in VSMCs through the reduction of the lncRNA NONHSAT 084969.2/nuclear factor-κB pathway [187]. BM-MSCs-derived exosomes also contribute to the prevention of calcification by transferring miR-15a/15b/16 and inhibiting their shared target gene NFATc3. This leads to the down-regulation of osteocalcin (OCN) expression, which in turn prevents the transformation of HA-VSMC into osteogenic cells [188].

### 8.5 Angiogenesis

MSC-derived EVs reportedly enhance the process of stroke healing by microvascular remodeling. MSC-EVs affect in vitro proliferation, migration, and tube formation of human cerebral microvascular ECs (hCMEC/D3), as well as their impacts on post-ischemic angiogenesis [189]. This concept is further strengthened by the fact that EVs generated by hypoxic MSCs are easily absorbed by ECs, resulting in increased proliferation, migration, and creation of blood vessels [190]. They have also been found to have a positive effect on EC senescence, promoting angiogenesis through the miR-146a/Src pathway. The presence of miR-146a has been shown to inhibit Src phosphorylation and its downstream targets VE-cadherin and Caveolin-1 [191]. Research has demonstrated that CPCs can release exosomes that contain a high concentration of miRNAs. These exosomes have been found to have anti-apoptotic and angiogenic (promoting blood vessel growth) effects. Furthermore, CPC exosomes secreted during hypoxia enhance EC tube formation and decrease the expression of pre-fibrotic genes in fibroblasts stimulated by transforming growth factor-β [192,193]. The exosomes derived from MCSs overexpressing stromal cell-derived factor 1 (SDF1) have the potential to be used as a therapeutic drug as the overexpression of SDF1 suppressed the programmed cell death and self-degradation of ischemic myocardial cells while stimulating the restoration of small blood vessels in the ECs of the heart [194]. When considered collectively, all of these findings point to the exosome bodies' potential for therapeutic use.

## 9 Human clinical trials of stem cell-derived exosomes

Stem cell-derived exosome treatment has bright future potential. Global market projections predict that the global exosome market will reach $6.8 billion [195], while the global stem cell market is expected to reach $56.15 billion by 2032 [196]. The data presented by the National Institutes of Health Guidelines for Human Stem Cell Research in 2022 primarily address ethical concerns and regulatory laws on human ESCs and iPSCs; reporting that in 2021, 90 human clinical trials were registered with over 3000 volunteers from 13 countries. In the past few years, there has been an upsurge in human trials using iPSCs [196]. Furthermore, some international organizations, such as the International Society for Stem Cell Research and the International Society for Extracellular Vesicles (ISEV) have put up regulations to tackle contentious challenges associated with stem cell and exosome research [79,197]. Although a universal agreement on how to translate and produce stem cells and exosomes is still obscure; these regulations aid in exploring these topics. A promising stem cell-based international standard prototype (ISO 24603) was issued in August 2022 that helped establish uniformity in the preliminary phases of exosome development for exosome therapies [198,199].

## 10 Potential safety concerns and regulatory challenges in standardizing exosome-based therapies

Despite encouraging therapeutic potential, exosome-based therapies cannot be employed clinically until biopharmaceutical safety precedents are established. The variability in manufacturing methods makes it difficult to standardize them, which results in a disunited regulatory framework. Moreover, their regulatory laws are intricate and differ among states due to their distinct intracellular mechanism of action. In several states, such as the US, Europe, Japan, South Korea, and Taiwan, regulatory bodies attempt to build a framework that strikes a balance between safety and efficacy. These states are keeping tabs on and providing pertinent, meaningful data to help future regulatory bodies standardize information on drug effects and cellular activities [200]. Moreover, the ISEV strives to establish safety standards [79], even though exosome treatment is still not clinically available due to a lack of established guidelines for exosome retrieval and a paucity of biopharmaceutical safety standards [201]. Furthermore, despite the growing availability of exosome-based therapeutic agents in private healthcare centers, the FDA has not approved any of them due to the absence of a unified regulatory framework [202]. Therefore, it is suggested that exosome-based therapeutics be subject to the same regulations as biological drugs and be authorized for use in clinical settings following the demonstration of their pharmacokinetics and therapeutic effectiveness as well as their comprehension at the molecular level.

The clinical implementation of stem cell-derived exosomal therapeutics depends on their mass production in GMP facilities. Nevertheless, there are still few GMP facilities available for exosome production, isolation, and quality assurance. Exosomes are isolated in laboratories via flask-based culture systems and ultracentrifugation techniques, which are tedious, time-consuming, and incompatible with the mass production of GMP-grade exosomes. On the other hand, for massive exosome production, hollow-fiber membranes and bioreactor systems are being investigated for filtration and cell culture, respectively. Besides, to reduce batch-to-batch variations, strict quality control is required. Therefore, methods are needed that can precisely and consistently characterize exosomes, such as hydrodynamic diameter, zeta potential, and exosome markers. Equipment like ZetaView, Amnis, ImageStream, and ONI Nanoimager can be used to characterize exosomes and could eventually take the role of more traditional methods like flow cytometry, dynamic light scattering, western blotting, and nanoparticle-tracking analysis. The heterogeneity of exosomes should also be taken into account for quality assurance, which relies on the isolation techniques and donor cell condition; with necessary standardizing operational protocols. Massive exosome production should be integrated with a fileable quality assurance system through the establishment of a simplified controlled operating system. With batch homogeneity and less physical labor, such a programmed and digitized method could further lower the price of exosome supplies [199].

## 11 Limitations and future perspective

Exosomes play a crucial role in the treatment of atherosclerosis and can be targeted for therapy or used as carriers for various payloads to cure atherosclerosis. Compared to other nanoparticles like liposomes and polymer nanoparticles, exosomes have significant advantages as a drug delivery medium. For instance, exosomes are nonimmunogenic because their composition closely resembles that of human cells, which reduces the likelihood of rejection [203]. Despite the promising potential of exosomes as therapeutic agents due to their ability to carry therapeutic compounds and their biocompatibility, there are still constraints regarding their manufacturing timeline and quantitative efficiency [82]. Moreover, since exosomes can carry a variety of substances, including proteins, lipids, and RNAs [204], it is challenging to pinpoint their precise impact on the targeted organ, e.g., the heart. This raises the dilemma of how to delineate the dominant effect of exosomes. It is crucial to think about whether the impact of a single molecule or molecules transported by an exosome may differ from the impact of the exosome itself. For instance, even though miRNAs have been shown to help restore heart function [[205], [206], [207]], superior technology is still required to identify the precise RNAs causing the practical improvement in end-organ performance.

The aforementioned safety issues and regulatory riddles, including but not limited to standardization, biopharmaceutical regulation, and particular clinical use of exosome treatment, can be resolved by comprehending the biological plausibility; and manufacturing and reproducibility of miRNA as molecular cargo [201]. The majority of research is directed towards miRNAs in exosomes, with less focus given to other small compounds. How minute medications placed within exosomes interact with other exosomal components before release from the vesicle remains unknown. To induce MCSs exosomes to express target proteins continuously and to gain an understanding of how they facilitate intercellular communication, appropriate methodologies must be developed. Moreover, exosomes derived from various sources exhibit notable distinctions and are susceptible to alterations in the microenvironment [208]. To meet therapeutic requirements, it is necessary to produce exosomes on a large scale while guaranteeing their functional integrity for the clinical translation of exosome therapy. Also, If genetic engineering is required to modify the exosomes on a wide scale, there is a tedious and expensive issue [94]. It is imperative to verify the safety and effectiveness of MSC-derived exosomes to improve their application in the treatment of atherosclerosis. Nevertheless, the majority of ongoing experiments are carried out on animals, while experiments conducted on humans are insufficient.

Improving the effectiveness of drug loading is a major challenge in the development of exosomes. One of the primary obstacles is that exosomes already carry cargo from their parent cells, so it can be difficult to introduce exogenous medications. Because of this, the loading capacity of exosomes is limited, which is why their drug-loading efficiency tends to be lower than that of liposomes [209]. Several studies have proven that the identical loading technique is employed to load cholesterol-modified siRNA to create engineered exosomes and anionic fusion liposomes. This is done to compare their respective abilities to carry substances. Engineered exosomes are unable to effectively deliver specific short RNAs, whereas anionic fusion liposomes can successfully induce the deletion of target genes by the use of siRNA [210].

Research is currently underway to develop exosome mimetics in order to address the constraint of the limited quantity of exosomes released by cells [211]. In order to address the issue of low efficiency in loading cargo into exosomes, a potential solution is to utilize optically reversible protein-protein interaction for protein loading, which can create a new type of exosomal protein carrier. Furthermore, the use of targeted therapeutics with exosomes can be accomplished through three methods. Firstly, targeting peptides can be added to the surface of exosomes. Secondly, specific genes can be transferred within exosomes to establish a therapeutic target in tumors. Lastly, exosomes containing tumor-associated antigens can be targeted [156]. By examining the characteristics of specific samples and application situations, we are confident that the deliberate use of isolation strategies, either alone or in combination, can effectively tackle the various obstacles encountered in contemporary exosome research [208]. Researchers have attempted to produce nanosized vesicles using nanovesicle biofabrication techniques. Additional research that concentrates on developing methods for large-scale synthesis or specific targeting of exosomes could enhance the effectiveness of employing exosomes for treating atherosclerosis [212].

When all of these factors are considered, it is necessary to make ongoing efforts to comprehend the precise mechanisms that underlie the therapeutic effects of exosomes, and consequently, the possibility of modifying or improving these mechanisms. It is also necessary to conduct additional research in order to determine the most effective method of administering exosomes, the recommended dosage, as well as their pharmacokinetics and biodistribution inside the body. One of the most important things that should be done is to test the modified exosomes for their effectiveness, immunogenicity, and overall safety [47,213].

## 12 Conclusion

Exosomes derived from stem cells have emerged as a highly promising therapeutic method for treating atherosclerosis. These exosomes have the potential to effectively regulate dyslipidemia, stabilize plaque, prevent plaque calcification, and promote plaque stability, thereby controlling atherosclerosis at every stage of its development. Although there are some challenges associated with using exosomes for the treatment of atherosclerosis, such as difficulties in isolating them, limited commercial availability, issues with drug loading, and a lack of sufficient research on human subjects, the potential benefits of this therapy are significant. With further research and development, exosome therapy has the potential to revolutionize the treatment of atherosclerosis and improve the lives of millions of people.

## CRediT authorship contribution statement

**Hassan Tariq:** Writing – original draft, Resources, Methodology, Investigation, Formal analysis, Data curation, Conceptualization. **Syeda Zunaira Bukhari:** Writing – original draft, Validation, Methodology, Investigation, Formal analysis. **Ruibing An:** Writing – review & editing, Validation, Project administration, Funding acquisition. **Jian Dong:** Writing – review & editing, Validation, Supervision, Project administration, Funding acquisition. **Ayesha Ihsan:** Writing – review & editing, Validation, Supervision, Project administration, Conceptualization. **Muhammad Rizwan Younis:** Writing – review & editing, Supervision, Project administration, Conceptualization, Funding acquisition.

## Declaration of competing interest

The authors declare that they have no known competing financial interests or personal relationships that could have appeared to influence the work reported in this paper.

## Data Availability

No data was used for the research described in the article.
